# Technical Acoustic Measurements Combined with Clinical Parameters for the Differential Diagnosis of Nonalcoholic Steatohepatitis

**DOI:** 10.3390/diagnostics13091547

**Published:** 2023-04-26

**Authors:** Yanan Zhao, Chen Qiu, Yiping Dong, Xuchu Wang, Jifan Chen, Jianting Yao, Yifan Jiang, Chao Zhang, Huifang Weng, Yajing Liu, Yik-Ning Wong, Pintong Huang

**Affiliations:** 1 Department of Ultrasound Medicine, The Second Affiliated Hospital of Zhejiang University School of Medicine, Hangzhou 310009, China; 2 Department of Laboratory, The Second Affiliated Hospital of Zhejiang University School of Medicine, Hangzhou 310009, China; 3 Canon Medical Systems China, Beijing 100015, China; 4 Binjiang Institute of Zhejiang University, Hangzhou 310053, China

**Keywords:** NASH, SWD, SWS, steatosis, nomogram

## Abstract

**Background and aim:** Diagnosing nonalcoholic steatohepatitis (NASH) is challenging. This study intended to explore the diagnostic value of multiple technical acoustic measurements in the diagnosis of NASH, and to establish a diagnostic model combining technical acoustic measurements with clinical parameters to improve the diagnostic efficacy of NASH. **Methods:** We consecutively enrolled 75 patients with clinically suspected nonalcoholic fatty liver disease (NAFLD) who underwent percutaneous liver biopsy in our hospital from June 2020 to December 2021. All cases underwent multiple advanced acoustic measurements for liver such as shear wave dispersion (SWD), shear wave speed (SWS), attenuation imaging (ATI), normalized local variance (NLV), and liver–kidney intensity ratio (Ratio) examination before liver biopsies. A nomogram prediction model combining the technical acoustic measurements and clinical parameters was established and the model is proposed to improve the diagnostic performance of NASH. **Results:** A total of 75 cases were included in this study. The classification of pathological grade for NASH was as follows: normal liver, (*n* = 15, 20%), nonalcoholic fatty liver (NAFL), (*n* = 44, 58.7%), and NASH, (*n* = 16, 21.3%). There were statistically significant differences in SWS (*p* = 0.002), acoustic coefficient (AC) (*p* = 0.018), NLV (*p* = 0.033), age (*p* = 0.013) and fasting blood glucose (Glu) (*p* = 0.049) between NASH and non-NASH. A nomogram model which includes SWS, AC, NLV, age and Glu was built to predict NASH, and the calibration curves showed good calibrations in both training and validation sets. The AUCs of the combined nomogram model for the training set and validation set were 0.8597 and 0.7794, respectively. **Conclusion:** There were statistically significant differences in SWS, AC, NLV, age and Glu between NASH and non-NASH. A nomogram model which includes SWS, AC, NLV, age and Glu was built to predict NASH. The predictive model has a higher diagnostic performance than a single factor model in the diagnosis of NASH and has good clinical application prospects.

## 1. Introduction

Nonalcoholic fatty liver disease (NAFLD) is one of the most common chronic liver diseases in the world, with reports suggesting that it affects 25% of the global population [1,2]. The NAFLD disease spectrum includes nonalcoholic fatty liver (NAFL), nonalcoholic steatohepatitis (NASH) and related liver cirrhosis. NASH is characterized by the presence of hepatic steatosis and liver injury such as ballooning degeneration and intralobular inflammation, and may lead to liver fibrosis or cirrhosis [3]. Approximately 20% to 40% of patients with NASH will develop advanced liver fibrosis, and are at risk for progressing to end-stage liver disease, hepatocellular carcinoma and extrahepatic complications [4,5,6,7].

Liver biopsy is currently the most reliable method for identifying NASH and fibrosis in NAFLD patients [3]. However, liver biopsy is limited by cost, sampling errors, and complications related to surgery. Many non-invasive methods aim to identify NAFLD patients with increased risk of disease progression. Shear wave elastography (SWE) or shear wave speed (SWS) can be used for liver stiffness assessment in NAFLD patients, and has good diagnostic performance in excluding severe fibrosis and liver cirrhosis [8,9]. The non-invasive assessment of NASH is a research hotspot, but currently there is no state-of-the-art imaging method. Shear wave dispersion (SWD) developed by Canon Medical Systems is used to evaluate the dispersion slope of shear wave, which indirectly reflects the viscosity of liver tissue, thereby predicting the degree of liver necrosis. Published research shows that SWD is significantly associated with lobular inflammation [10]. SWD has higher diagnostic power than liver stiffness values in predicting liver allograft injury [11].

The attenuation imaging (ATI) technique quantifies hepatic steatosis by calculating the attenuation of ultrasound beam in the liver tissue and has shown good clinical diagnostic performance in published studies [10,12,13,14]. The normalized local variance (NLV) technique can assess hepatic steatosis by calculating the difference between the theoretical and actual echo amplitude distributions of the liver parenchyma [15]. Our preliminary clinical study has shown that NLV and the standard deviation of NLV (NLV-SD) have good diagnostic performance in detecting different degrees of hepatic steatosis with high reproducibility [16]. The liver–kidney intensity ratio (Ratio) has been used as a quantitative method for the detection of hepatic steatosis. Although its overall performance is good, it varies widely in terms of diagnostic cutoffs and accuracy [17,18].

The purpose of this study was to explore the value of technical acoustic measurements such as SWS, SWD, ATI, NLV and Ratio in the diagnosis of NASH, using histopathology as the gold standard. A proposed non-invasive diagnostic model, which combines the technical acoustic measurements and clinical parameters, was established to further improve the diagnostic value of NASH.

## 2. Materials and Methods

### 2.1. Patients

This prospective, single-center study was approved by the Institutional Review Board of the Second Affiliated Hospital of Zhejiang University School of Medicine. Written informed consent was obtained from all patients. All methods were performed in accordance with the Declaration of Helsinki. Patients who were suspected to have NAFLD and were referred for liver biopsy to evaluate the etiology and disease activity were consecutively enrolled from the department of hepatology between July 2020 and December 2021. The inclusion criteria were as follows: individuals aged ≥20 years; patients without a history of alcohol use (pure alcohol below 30 g/day for males, 20 g/day for females); hepatic steatosis detected by B-mode ultrasound (US) or computed tomography (CT). The exclusion criteria were the following: patients with viral hepatitis, excessive alcohol consumption, malignant liver tumor, choledocholithiasis, and jaundice; patients with primary biliary cholangitis, primary sclerosing cholangitis, and autoimmune hepatitis; patients with comorbidities (imposing prolonged fasting or total parenteral nutrition); patients taking any medication that may reduce blood glucose or cholesterol levels.

### 2.2. ATI, NLV and Ratio Examination

**Figure 1:**
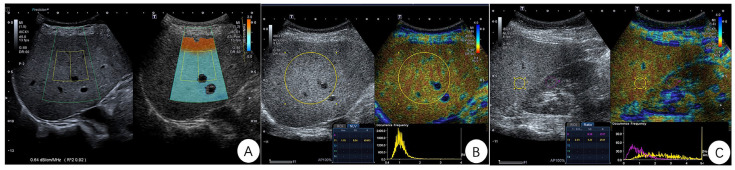
Representative images of the ATI, NLV, and Ratio examination in one patient. (**A**) ATI image: The ultrasound (US) image is shown on the left and the same US image overlaid with the attenuation map is shown on the right. The attenuation coefficient (AC) is shown to be 0.64 dB/cm/MHz, and the coefficient of determination (R^2^) is 0.92. (**B**) NLV image: A 50 mm ROI is placed in the liver parenchyma and 10 mm below the liver capsule. The average value (Ave) of NLV is 1.15 and the standard deviation (SD) is 0.54. (**C**) Ratio image: A 10 mm circular ROI is free of vessels or artifacts within the liver parenchyma, and a 10 mm circular ROI is placed within the right kidney cortex which is free of fat, large vessels and renal pyramids. The liver–kidney intensity ratio (Ratio) of 2.51 and SD of 1.27 are displayed at the bottom of the image.

All US examinations were conducted by one radiologist (with six years of experience in abdominal US imaging) using an US scanner with a convex transducer (PVI-475BX, 4 MHz; Aplio i900; Canon Medical Systems, Tochigi, Japan) prior to liver biopsy. The patient was in the supine position and the right arm was extended above the head to stretch the intercostal muscles and to obtain the proper scanning window during the examination. First, liver parenchyma was evaluated on B-mode images to detect any focal liver lesions and to measure the vertical distance of the liver capsule from the skin surface. After that, ATI mode was activated, and examinations were performed in the right lobe of the liver through an intercostal window with the transducer perpendicular to the skin surface while the patient held his or her breath. A fan-shaped sample box was placed onto the liver right lobe parenchyma, and a measurement ROI (region of interest), 2 cm wide × 4 cm high, was placed in the center of the sample box, avoiding multiple reflection artifacts and large vessel areas. The AC (acoustic coefficient) value was displayed in dB/cm/MHz at the bottom of the image. The R^2^ (coefficient of determination) value indicates the reliability of the results. R^2^ ≥ 0.8 was considered to be reliable. Five AC measurements were obtained, and the median value was chosen for analysis. Then, NLV mode was activated, a 50 mm circular ROI was placed in the middle of the US image and 10 mm under the liver capsule. The NLV value was automatically calculated and displayed at the bottom of the image. NLV examination was performed from four different US images, and the median value was obtained for analysis. Next, we kept the transducer in the right intercostal space, displaying both liver and kidney clearly, then we switched to intensity analysis mode. We placed a 10 mm circular ROI which was free of vessels or artifacts within the liver parenchyma, and another 10 mm circular ROI was placed within the right kidney cortex that was free of fat, large vessels and renal pyramids. The liver–kidney intensity ratio (Ratio) was displayed at the bottom of image. We repeated the measurement four times, and the median value was used for analysis. The representative images of the ATI, NLV and Ratio examinations are shown in Figure 1.

### 2.3. SWS and SWD Examination

**Figure 2:**
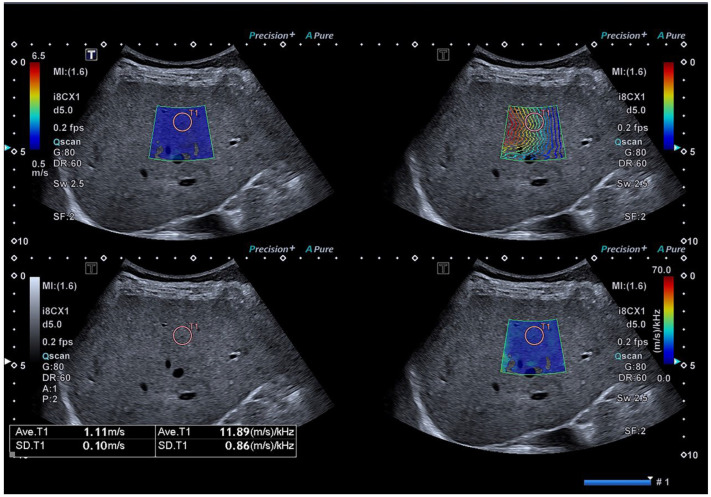
Representative image of the SWD/SWS examination. A shear wave elasticity map is on the upper left, shear wave propagation map is on the upper right, normal US image is on the lower left and shear wave dispersion map is on the lower right. A 10 mm circular ROI is placed in the liver parenchyma free of large hepatic vessels under the guidance of the propagation map. A shear wave speed (SWS) value of 1.11 m/s and shear wave dispersion (SWD) value of 11.89 (m/s)/kHz are shown at the bottom of image.

The SWS and SWD examination was conducted in the liver lobe through the intercostal space. A 4 × 3 cm sample box free of reverberation artifacts was placed on grayscale imaging at least 1 cm beneath the liver capsule according to the liver elastography guideline [19]. After acoustic radiation force excitation, shear waves were induced and the US system automatically displayed the B-mode image, shear wave propagation map, SWS image and SWD image (Figure 2). We placed a 10 mm circular ROI in the liver parenchyma free of large hepatic vessels under the guidance of the propagation map. The SWS value and SWD value were displayed at the bottom of image. The median values of ten measurements of SWS and SWD were chosen for further analysis. We defined a reliable measurement as an interquartile range (IQR)/median value of less than 30% from the ten measurements.

### 2.4. Biochemical Examination and Clinical Data Collection

We documented patients’ medical history and blood test data before the liver biopsy. The clinical data included age, gender, height (cm), weight (kg), waist circumference (cm), BMI (kg/m^2^), the presence of diabetes mellitus or hypertension. Fasting blood test items included platelet counts (PLT) (10^9^/L), aspartate aminotransferase (AST) (IU/L), alanine aminotransferase (ALT) (IU/L), gamma-glutamyl transpeptidase (GGT) (IU/L), fasting blood glucose (Glu) (mmol/L), triglycerides (TG) (mmol/L), total cholesterol (TC) (mmol/L), total bilirubin (TBIL) (μmol/L), alkaline phosphatase (ALP) (U/L), albumin (ALB) (g/dL), low density lipoprotein cholesterol (LDL-C) (mmol/L), high density lipoprotein cholesterol (HDL-C) (mmol/L), plasma urea nitrogen (BUN) (mmol/L) and uric acid (UA) (μmol/L). Serum cytokeratin-18 (CK-18), human soluble apoptosis-related factor (sFAS) and fibroblast growth factor 21 (FGF-21) were detected by the ELISA kit (Shanghai Enzyme-linked Biotechnology Co., Ltd.).

### 2.5. Histopathological Examination

A percutaneous liver biopsy was performed within 2 weeks after US examinations using a semi-automatized needle (NS18/16, NS16/16, GALLINI S R L.). The biopsy was performed in liver segment V or VIII. A liver specimen of more than 1.5 mm with at least nine portal tracts was considered adequate for the evaluation. Liver biopsy specimens were fixed in formalin and embedded in paraffin. Subsequently, 5 μm-thick slices were cut and stained with hematoxylin–eosin and Masson’s trichrome. All histopathological assessments were analyzed by two pathologists (with 15 and 5 years of experience in liver pathology) who were blinded to the US examination results. Inconsistent results were agreed upon by two pathologists after discussion. The NAFLD Activity Score (NAS) was used to evaluate the pathological parameters of NAFLD (steatosis, intralobular inflammation and ballooning) [20]. The degree of steatosis (S) was graded on a four-point scale as follows: S0 (<5%, none), S1 (5–33%, mild), S2 (>33–66%, moderate) and S3 (>66%, severe). Lobular inflammation (I) was graded from score 0 to 3 as follows: I0 (no foci), I1 (<2 foci per 200×filed), I2 (2–4 foci per 200×filed), I3 (>4 foci per 200×filed). Hepatocyte ballooning degeneration (B) was graded from score 0 to 2 as follows: B0 (none), B1 (few balloon cells), B2 (many cells or prominent ballooning). NASH and NAFL were defined by Matteoni classification [21], as follows: Type 1, only hepatic steatosis; Type 2, hepatic steatosis with intralobular inflammation; Type 3, hepatic steatosis with ballooning degeneration; Type 4, hepatic steatosis with ballooning degeneration and Mallory body or fibrosis. Type 1 and Type 2 were defined as NAFL, Type 3 and Type 4 were defined as NASH. The fibrosis stage (F) was evaluated on a five-point scale from F0 to F4 according to Brunt Classification [22], as follows: F0 (no fibrosis), F1 (fibrosis near lobule center), F2 (fibrosis near lobule center and periportal fibrosis), F3 (bridging fibrosis) and F4 (cirrhosis).

### 2.6. Statistical Analysis

A statistical analysis was performed using SPSS software version 17 (IBM Corp., Armonk, NY, USA), MedCalc software version 12.1.00 (MedCalc Software, Mariakerke, Belgium) and package R (3.3.3 version, https://www.r-project.org, 6 March 2017). Continuous data were expressed as mean ± standard deviation or median ± interquartile range according to the normality of the data; count data were presented as the absolute number or percentage. Spearman’s rank correlation coefficient was used to evaluate the correlation of pathological parameters with both technical acoustic measurements and clinical related parameters. Continuous variables were compared using the Kruskal–Wallis test, and categorical variables were evaluated using the chi-square test or the Fisher’s exact test. The differences in various technical acoustic measurements and clinical parameters between the NASH and non-NASH groups were compared by an independent samples t test or Mann–Whitney U test. Binary logistic regression analysis was performed for NASH data as the dependent variable, and the variables with statistical difference obtained from the univariate analysis as independent variables to obtain significant factors affecting NASH. The diagnostic performance of different parameters for the differentiation of NASH was calculated by the receiver operating characteristic (ROC) curve analysis. Binary logistic regression for LASSO regression was performed using the “glmnet” package. Nomogram development and calibration curve plotting were developed using the “rms” package. Decision curve analysis was performed using the “rmda” package. All significance tests were conducted two-sided and descriptive levels (*p* values) lower than 0.05 were considered statistically significant.

## 3. Results

### 3.1. Baseline Characteristics

During the study period, a total of 87 consecutive patients with fatty liver revealed by conventional US or abdominal CT were referred for a liver biopsy at our institution. A total of 12 patients with chronic hepatitis B were excluded from the study and our final study population comprised a total of 75 patients with 9 cases of donors for liver transplantation. The median age and body mass index (BMI) were 54.0 years (40.0–60.0) and 25.8 kg/m^2^ (23.7–28.6), respectively. The participants’ baseline demographic, biochemical, and histological data are summarized in Table 1.

### 3.2. Correlation between Pathological Parameters versus Technical Acoustic Measurements and Clinical Parameters

In pathological grading, the distribution of hepatic steatosis grade on histopathology was 15/41/13/6 for none (<5%)/mild (5–33%)/moderate (>33–66%)/and severe (>66%) steatosis, respectively. The intralobular inflammation was graded as follows: I0, *n* = 30 (40.0%), I1, *n* = 36 (48.0%), I2, *n* = 7 (9.3%), I3, *n* = 2 (2.7%). The ballooning grades were as follows: B0, *n* = 59 (78.7%), B1, *n* = 13 (17.3%), B2, *n* = 3 (4.0%). The fibrosis was graded as follows: F0, *n* = 48 (64.0%), F1, *n* = 20 (26.7%), F2, *n* = 6 (8.0%), F3, *n* = 1 (1.3%), F4, *n* = 0 (0%). The pathological grade of NASH was as follows: normal liver, *n* = 15 (20%), NAFL, *n* = 44 (58.7%), NASH, *n* = 16 (21.3%).

Supplementary Table S1 shows the correlation between acoustic technical parameters and pathological parameters. The AC value (r = 0.519), Ratio value (r = 0.285) and SWS value (r = 0.295) showed significant positive correlations with hepatic steatosis; the NLV value (r = −0.391) and NLV-SD value (r = −0.356) showed significant negative correlations with hepatic steatosis. The AC value showed a significant positive correlation with intralobular inflammation (r = 0.353). The NLV value (r = −0.379) and NLV-SD value (r = −0.326) showed significant negative correlations with intralobular inflammation. The SWS (r = 0.315) and AC (r = 0.290) values showed significantly positive correlations with hepatocyte ballooning degeneration. The NLV value (r = −0.250) showed a significantly negative correlation with hepatocyte ballooning degeneration. The SWS (r = 0.306) value showed significant positive correlations with liver fibrosis grade. The SWS (r = 0.312) and AC (r = 0.286) values showed significant positive correlations with NASH. The NLV (r = −0.254) value showed a significant negative correlation with NASH. Supplementary Table S2 shows the differences in various technical acoustic measurements and clinical parameters between non-NASH and NASH patients. There were statistically significant differences in SWS (*p* = 0.002), AC (*p* = 0.018), NLV (*p* = 0.033), age (*p* = 0.013) and Glu (*p* = 0.049) between NASH and non-NASH groups. There was no statistically significant difference in SWD between NASH and non-NASH.

### 3.3. Influencing Factors of AC, NLV, NLV-SD, Ratio, SWE and SWS

The factors affecting the SWS AC, NLV, NLV-SD and Ratio are shown in the Supplementary Tables S1–S7. Fibrosis grade (*p* = 0.005) and AST (*p* = 0.001) were the significant influencing factors of the SWS value in multivariate regression analysis (Supplementary Table S3). Hepatic steatosis (*p* < 0.001) and diabetes were the significant influencing factors of the AC value in multivariate regression analysis (Supplementary Table S4). Intralobular inflammation (*p* = 0.041) and waist circumference (*p* = 0.012) were the significant influencing factors of the NLV value in multivariate regression analysis (Supplementary Table S5). The degree of steatosis (*p* = 0.026) and waist circumference (*p* = 0.006) were the significant influencing factors of the NLV-SD value in multivariate regression analysis (Supplementary Table S6). Hepatic steatosis was the significant influencing factor of the Ratio value in univariate regression analysis (Supplementary Table S7).

### 3.4. The Diagnostic Performance of SWS, AC and NLV Values in Differentiating NASH from Non-NASH

The AUCs (area under curves) of the SWS value for the diagnosis of NASH were 0.719 (95% CI 0.604–0.817), with sensitivity of 62.5% (95% CI 35.4–84.8%) and specificity of 81.4% (95% CI 69.1–90.3%). The AUCs of AC and NLV values for the diagnosis of NASH were 0.702 (95% CI 0.582–0.805) and 0.676 (95% CI 0.554–0.782), respectively, with sensitivities of 93.3% (95% CI 68.1–99.8%) and 87.5% (95% CI 61.7–98.4%) and specificities of 44.6% (95% CI 31.3–58.5%) and 54.6% (95% CI 40.6–68.0%), respectively (Table 2).

### 3.5. Establishment of a Nomogram Prediction Model Combining Technical Acoustic Measurements and Clinical Parameters for the Combined Diagnosis of NASH

**Figure 3:**
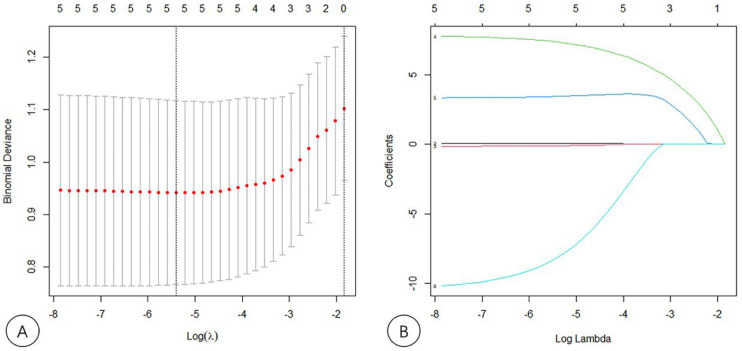
Risk predictor selection using the LASSO logistic regression model. (**A**) Tunning parameter (λ) selection in the LASSO model using ten-fold cross-validation via the minimum criterion. The optimal value of the LASSO tuning parameter (λ) is indicated by the dotted vertical lines. (**B**) LASSO coefficient profiles of five radiomics features. A coefficient profile plot was generated versus the selected logλ using ten-fold cross-validation. Five radiomics features with non-zero coefficients were selected.

**Figure 4:**
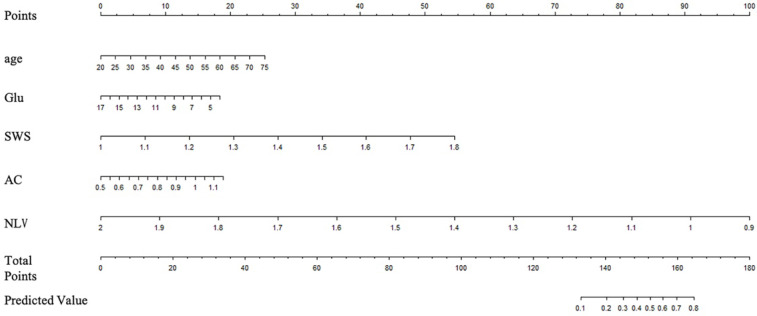
A combined nomogram model which comprises age, Glu, SWS, AC and NLV is developed from the training set.

**Figure 5:**
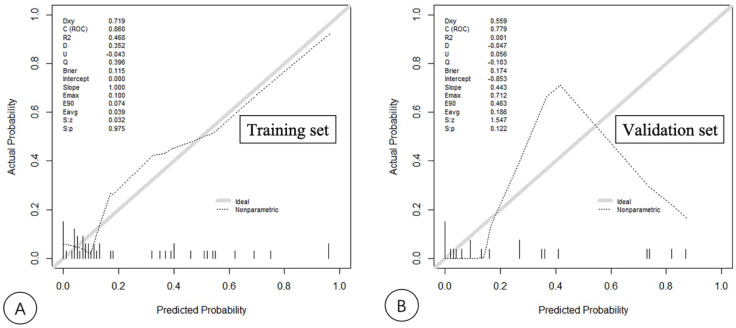
Calibration curves for the combined nomogram model in the training (**A**) and validation (**B**) sets. Calibration curves indicate the goodness-of-fit of the nomogram. The diagonal line represents the perfect match between the actual (Y-axis) and nomogram-predicted (X-axis) probabilities. A closer distance between the two curves means that a higher accuracy can be obtained.

**Figure 6:**
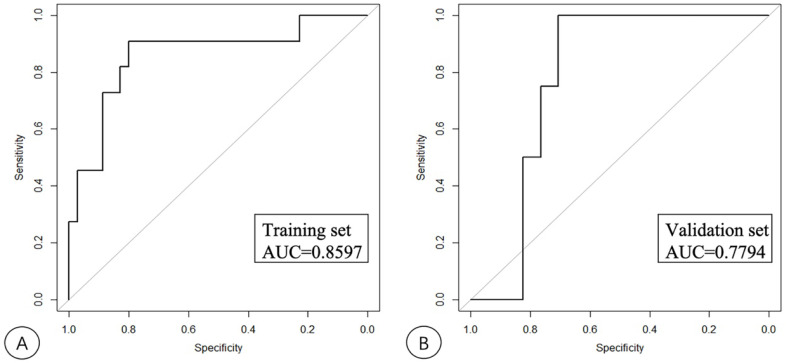
The receiver operating characteristic (ROC) curves of the combined nomogram model in the training (**A**) and validation (**B**) sets.

According to the results from the univariate analysis of NASH, 75 patients were randomly divided into the training and validation sets according to the ratio of 7:3 in this study. The clinical factors of the training and validation sets are shown in Table 3. SWS, AC, NLV, age and Glu were screened out by LASSO regression as the key factors for the diagnosis of NASH, and a nomogram model which includes SWS, AC, NLV, age and Glu was built to predict NASH. The key factors for the diagnosis of NASH, SWS, AC, NLV, age and Glu were included to construct a nomogram model through logistic regression. The variable selection is shown in Figure 3; the model establishment is shown in Figure 4. The calibration curves showed good calibrations in both training and validation sets (Figure 5). The AUCs of the combined nomogram model in the training and validation sets were 0.8597 and 0.7794, respectively (Figure 6). The combined nomogram model had a higher overall net benefit than the single factor model within most reasonable threshold probabilities for the identification of NASH based on decision curve analysis (Supplementary Figure S1).

## 4. Discussion

The differential diagnosis of simple hepatic steatosis and NASH is the key in determining the treatment and follow-up schedules of NAFLD patients. NASH patients are at risk for progression to liver fibrosis, cirrhosis, and liver cancer [23]. Therefore, there is significant clinical value in identifying NASH patients and implementing lifestyle changes and drug therapy [24]. In this study, a nomogram prediction model was established that combined technical acoustic measurements with clinical parameters, which improved the diagnostic accuracy of NASH. Our preliminary research showed that NASH was significantly positively correlated with SWS and AC values, and significantly negatively correlated with NLV values. SWS, AC, NLV, age and Glu were significantly different between NASH and non-NASH groups. The nomogram was constructed combining technical acoustic measurements and clinical parameters, and its AUCs were 0.8597 and 0.7794 in the training and validation sets, respectively. The combined nomogram model had a higher overall net benefit than the single factor model within most reasonable threshold probabilities for the identification of NASH.

Ultrasound quantitative technology has shown high sensitivity and feasibility in detecting hepatic steatosis. However, it has been difficult to break through in the differential diagnosis of NASH. Some studies suggested that a controlled attenuation parameter (CAP, Fibroscan, France) is less accurate for NASH detection and may be affected by advanced fibrosis [25]. Another study of 358 NASH subjects showed that TE was only moderately accurate in diagnosing NASH with the combined nomogram model (LSM, Fibroscan, France), and the CAP model had a diagnostic power of 0.71 [26]. Published clinical studies have shown that SWD can predict necroinflammation activity [10], and SWD has higher diagnostic performance than liver stiffness values in the detection of liver allograft injury [11]. Our study found that SWD had no statistically significant difference in intralobular inflammation, ballooning degeneration, fibrosis grade and the differential diagnosis of NASH, which was consistent with a few previous studies [27]. However, there are relatively few studies on SWD, and the results are inconsistent. Some studies [28] showed that SWS was better than SWD for predicting the degree of fibrosis, and SWD was more useful than SWS for predicting the degree of necroinflammation. Considering the relatively small number of NASH cases in this study (21.3%, 16/75), the proportion of ballooning degeneration related to NASH was relatively small (21.3%, 16/75). Moreover, this study did not further differentiate the severity of NASH, which may be an important factor for the negative result of SWD. Further research should increase the sample size, especially the proportion of NASH patients, for a more detailed analysis.

This study also found that NASH was positively correlated with the SWS and AC values, but negatively correlated with NLV values. SWS, AC and NLV values had moderate diagnostic performance in the diagnosis of NASH. SWS was a diagnostic predictor of NASH in the multivariate regression analysis. Previous animal studies [29] indicated that SWS can be used to assess disease phenotype and progression in preclinical models of NAFLD/NASH. SWS has been proven to have a high diagnostic value in the stage of liver fibrosis, and has been recommended in the non-invasive assessment of liver fibrosis by major guidelines and expert opinions [19,30,31]. This study further explored the value of SWS in the differential diagnosis of NASH. The main pathological features of NASH included hepatic ballooning degeneration, and inflammatory cell infiltration caused by fat accumulation. In our study, the degree of NASH with liver fibrosis was mostly in the F1-F2 stage, and rarely reached the F3-F4 stage, which indicated that the SWS value increased with the aggravation of inflammatory activity. Our preliminary study showed that the AC value and NLV value present moderate diagnostic performance in the differential diagnosis of NASH and non-NASH. However, hepatic steatosis is a significant factor affecting AC value, and intralobular inflammation is a significant factor affecting NLV value in the multivariate regression analysis. Therefore, further research is needed to explore the diagnosis value of hepatic steatosis and intralobular inflammation in NASH.

In this study, a nomogram combining technical acoustic measurements and clinical parameters was established to predict NASH. The nomogram contained SWS, AC, NLV, age and Glu, and the model obtained good diagnostic performance in the differential diagnosis of NASH. The AUC of nomogram reached 0.8597 in the training set, and 0.7749 in the validation set, which was higher than the diagnostic performance of a single indicator. Previous studies have proven that NASH is strongly associated with obesity, dyslipidemia, type 2 diabetes and metabolic syndrome [32]. Therefore, some relevant clinical parameters such as BMI, TG, TC or PLT cannot be excluded from the nomogram. The result in our study shows that these parameters had no significant difference in the diagnosis of NASH, which is probably due to the small sample size. Further research studies should increase the sample size in order to assess if additional clinical parameters should be included in the nomogram.

This study has certain limitations. First, this study is a single-center study with a small sample size, which leads to a bias in the selection of clinical parameters. Multi-center studies should be further carried out and the sample size should be expanded to explore the diagnostic performance of technical acoustic measurements and clinical parameters for NASH. Secondly, the number of NASH cases in this study was small and the degree of NASH was not distinguished. Preliminary research showed that several technical acoustic measurements are expected to have a diagnostic value in the diagnosis of NASH; the matter of whether they can identify the severity of NASH needs further research. Finally, the parameters in the nomogram in this study were parameters that were statistically significant in the univariate analysis. In future, multivariate analyses can be performed to obtain more variables with statistical differences by expanding the sample size, and a nomogram can be established to make it closer to clinical practical application.

## 5. Conclusions

There were statistically significant differences in SWS, AC, NLV, age and Glu between NASH and non-NASH patients. SWS is an independent influencing factor of NASH. A nomogram model which includes SWS, AC, NLV, age and Glu was built to predict NASH. The predictive model has a higher diagnostic performance than a single factor model in the diagnosis of NASH, and has a good clinical application prospect.

## Figures and Tables

**Table 1:** Baseline characteristics of the 75 patients with NAFLD.

Parameters	Patients (*n* = 75)
Age (years, median, IQR)	54.0 (40.0–60.0)
Sex (*n*, male:female)	35:40
Diabetes (*n*, yes:no)	11:64
Hypertension (*n*, yes:no)	36:39
BMI (kg/m^2^, median, IQR)	25.8 (23.7–28.6)
Waist circumference (cm, mean ± SD)	94.5 ± 10.0
**Liver biochemistry**	
AST (IU/L, median, IQR)	24.0 (19.0–29.0)
ALT (IU/L, median, IQR)	24.0 (19.0–40.0)
GGT (IU/L, median, IQR)	31.0 (19.8–49.8)
ALP (IU/L, mean ± SD)	84.4 ± 24.1
TBIL (μmol/L, median, IQR)	12.9 (9.1–15.2)
**Lipid profile**	
TC (mg/dl, mean ± SD)	5.3 ± 1.1
HDL-C (mg/dl, median, IQR)	1.2 (1.0–1.3)
LDL-C (mg/dl, mean ± SD)	3.0 ± 0.7
TG (mg/dl, median, IQR)	1.8 (1.3–2.3)
ALB (g/dL, mean ± SD)	43.4 ± 2.9
PLT (×10^9^/L, median, IQR)	213.0 (180.8–257.8)
Glu (mg/dl, median, IQR)	5.4 (5.0–6.0)
BUN (mmol/L, mean ± SD)	4.9 ± 1.3
UA (μmol/L, median, IQR)	344.0 (300.0–442.5)
Depth (cm)	1.8 (1.6–2.1)
**Degree of steatosis (%)**	
S0 (none, <5%)	15 (20.0)
S1 (mild, 5–33%)	41 (54.7)
S2 (moderate, >33–66%)	13 (17.3)
S3 (severe, >66%)	6 (8.0)
**Intralobular Inflammation**	
I0 (None)	30 (40.0)
I1 (Mild)	36 (48.0)
I2 (Moderate)	7 (9.3)
I3 (Severe)	2 (2.7)
**Ballooning Degeneration**	
B0 (None)	59 (78.7)
B1 (Few balloon cells)	13 (17.3)
B2 (Many cells/ prominent ballooning)	3 (4.0)
**Grade of fibrosis (%)**	
F0	48 (64.0)
F1	20 (26.7)
F2	6 (8.0)
F3	1 (1.3)
F4	0 (0)
**Pathological diagnosis**	
Normal liver	15 (20)
NAFL	44 (58.7)
NASH	16 (21.3)

BMI, body mass index; AST, aspartate aminotransferase; ALT, alanine aminotransferase; GGT, gamma-glutamyl transpeptidase; ALP, alkaline phosphatase; TBIL, total bilirubin; TC, total cholesterol; HDL-C, high density lipoprotein cholesterol; LDL-C, low density lipoprotein cholesterol; TG, triglycerides; ALB, albumin; PLT, platelet counts; Glu, fasting blood glucose; BUN, plasma urea nitrogen; UA, uric acid.

**Table 2:** The diagnostic performance of SWS, AC and NLV values in detecting NASH and non-NASH patients.

Parameters	Cutoff Value	AUC (95% CI)	Sensitivity (%) (95% CI)	Specificity (%) (95% CI)	PLR (95% CI)	NLR (95% CI)
SWS	>1.3	0.719 (0.604, 0.817)	62.5 (35.4, 84.8)	81.4 (69.1, 90.3)	3.35 (1.7, 6.4)	0.46 (0.2, 0.9)
AC	>0.67	0.702 (0.582, 0.805)	93.3 (68.1, 99.8)	44.6 (31.3, 58.5)	1.69 (1.3, 2.2)	0.15 (0.02, 1.0)
NLV	≤1.105	0.676 (0.554, 0.782)	87.5 (61.7, 98.4)	54.6 (40.6, 68.0)	1.92 (1.4, 2.7)	0.23 (0.06, 0.9)

AUC, area under curves; SWS, shear wave speed; AC, acoustic coefficient; NLV, normalized local variance; PLR, positive likelihood ratio; NLR, negative likelihood ratio.

**Table 3:** The clinical factors of the training and validation sets.

Parameters	Training Set (*n* = 52)	Validation Set (*n* = 13)	*p* Value
age	51.8 ± 11	50.0 ± 15	0.63
Glu	6.21 ± 2.36	5.45 ± 0.77	0.06
SWS	1.26 ± 0.14	1.27 ± 0.09	0.62
AC	0.75 ± 0.12	0.76 ± 0.14	0.76
NLV	1.13 ± 0.14	1.17 ± 0.12	0.35

Glu, fasting blood glucose; SWS, shear wave speed; AC, acoustic coefficient; NLV, normalized local variance.

## Data Availability

The datasets used and/or analyzed during the current study are available from the corresponding author upon reasonable request.

## Supplementary Materials

The following supporting information can be downloaded at: https://www.mdpi.com/article/10.3390/diagnostics13091547/s1.
