# Supplementary material for: Technical Acoustic Measurements Combined with Clinical Parameters for the Differential Diagnosis of Nonalcoholic Steatohepatitis
Source: Diagnostics (Basel). 2023 Apr 26;13(9):1547. doi: 10.3390/diagnostics13091547 (PMC10177914; doi:10.3390/diagnostics13091547)
Supplement: Supplementary file 1 [file diagnostics-13-01547-s001.zip › diagnostics-2312142-supplementary.pdf]

A diagnostic model of technical acoustic measurements combined with clinical parameters for differential diagnosis of nonalcoholic steatohepatitis

Sup Table S1 Correlation of liver pathological parameters with technical acoustic measurements and clinical parameters

**Pathological parameters**	SWS	SWD	AC	NLV	NLV-SD	Ratio
Steatosis	**0.295**	0.130	**0.519**	**-0.391**	**-0.356**	**0.285**
p value	0.010*	0.267	<0.001*	0.001*	0.002*	0.016*
Intralobular Inflammation	0.217	0.149	**0.353**	**-0.379**	**-0.326**	0.174
p value	0.062	0.202	0.003*	0.001*	0.006*	0.146
Ballooning Degeneration	**0.315**	0.102	**0.290**	**-0.250**	-0.216	0.161
p value	0.006*	0.386	0.014*	0.035*	0.070	0.599
Fibrosis	**0.306**	0.117	0.191	-0.199	-0.192	-0.124
p value	0.008*	0.317	0.111	0.097	0.108	0.304
NASH	**0.312**	0.108	**0.286**	**-0.254**	-0.221	0.164
p value	0.007*	0.358	0.015*	0.032*	0.064	0.173

Sup Table S1（continued）

**Pathological parameters**	Age	AST	ALT	GGT	TBIL	ALB	BUN
Steatosis	0.175	**0.450**	**0.520**	**0.301**	**0.255**	**0.280**	**-0.233**
p value	0.132	<0.001*	<0.001*	0.009*	0.027*	0.015*	0.044*
Intralobular	**0.312**	**0.458**	**0.330**	**0.246**	**0.231**	0.142	0.000

Inflammation							
p value	0.007*	0.000*	0.004*	0.035*	0.046*	0.224	1.000
Ballooning Degeneration	**0.299**	0.213	0.107	0.191	**0.238**	0.039	-0.035
p value	0.009*	0.067	0.363	0.104	0.040*	0.738	0.764
Fibrosis	**0.347**	**0.244**	**0.287**	0.197	0.067	0.011	-0.148
p value	0.002*	0.035*	0.012*	0.093	0.569	0.924	0.205
NASH	**0.287**	0.206	0.091	0.192	0.226	0.034	-0.050
p value	0.012*	0.076	0.437	0.101	0.051	0.773	0.672

SWS, shear wave speed; SWD, shear wave dispersion; AC, acoustic coefficient; NLV, normalized local variance; NLV-SD, standard deviation of NLV; Ratio, liver-kidney intensity ratio; NASH, nonalcoholic steatohepatitis; AST, aspartate aminotransferase; ALT, alanine aminotransferase; GGT, gamma-glutamyl trans-peptidase; TBIL, total bilirubin; ALB, albumin; BUN, plasma urea nitrogen.

Sup Table S2 Differences of various technical acoustic measurements and clinical parameters between non-NASH and NASH groups

**Parameters**	**non-NASH (n=59)**	**NASH (n=16)**	**Z/t value**	**p value**
SWS	1.24	1.34	-3.275†	**0.002***
SWD	11.35	11.85	-1.019†	0.311
AC	0.72	0.81	-2.433†	**0.018***
NLV	1.12	1.07	-2.127$^{\&}$	**0.033***
NLV-SD	0.43	0.29	-1.900$^{\&}$	0.057
Ratio	2.00	2.38	-1.362$^{\&}$	0.173
age	53.0	57.5	-2.472$^{\&}$	**0.013***
WC	94.4	95.2	-0.245†	0.807
BMI	25.3	26.2	-0.168$^{\&}$	0.866
PLT	226.5	217.4	0.487†	0.628
AST	23.0	24.0	-1.775$^{\&}$	0.076
ALT	24.0	25.5	-0.783$^{\&}$	0.434
Glu	5.29	5.61	-1.966$^{\&}$	**0.049***
TG	1.94	1.82	0.553†	0.582
TC	5.28	5.50	-0.701†	0.485
GGT	27.0	42.0	-1.641$^{\&}$	0.101
TBIL	11.7	24.9	-1.947$^{\&}$	0.052
ALP	84.3	84.4	-0.009†	0.993
ALB	43.4	43.7	-0.358†	0.721
LDL-C	3.0	2.8	1.041†	0.301

HDL-C	1.15	1.14	-0.420[&]	0.674
BUN	4.9	4.8	0.091[†]	0.928
UA	342.0	356.0	-0.800[&]	0.424
Distance	1.76	1.97	-1.552[&]	0.121
CK-18	7.8	6.2	-0.738[&]	0.461
sFAS	623.9	634.2	-0.344[†]	0.731
FGF-21	30.5	21.6	-1.476[†]	0.140

[&]Mann-Whitney U test; [†]independent sample t test; [*]$p<0.05$

NASH, nonalcoholic steatohepatitis; SWS, shear wave speed; SWD, shear wave dispersion; AC, acoustic coefficient; NLV, normalized local variance; NLV-SD, standard deviation of NLV; Ratio, liver-kidney intensity ratio; WC, waist circumference; BMI, body mass index; PLT, platelet counts; AST, aspartate aminotransferase; ALT, alanine aminotransferase; Glu, fasting blood glucose; TG, triglycerides; TC, total cholesterol; GGT, gamma-glutamyl transpeptidase; TBIL, total bilirubin; ALP, alkaline phosphatase; ALB, albumin; LDL-C, low density lipoprotein cholesterol; HDL-C, high density lipoprotein cholesterol; BUN, plasma urea nitrogen; UA, uric acid; Distance, distance from skin to liver capsule; CK-18 (cytokeratin-18), sFAS (human soluble apoptosis related factor), FGF-21 (fibroblast growth factor 21)

Sup Table S3 Factors associated with SWS value

Characteristic	Univariate Analysis			Multivariate Analysis		
	Coefficient	95% CI	p value	Coefficient	95% CI	p value
Degree of steatosis	0.046	0.013-0.079	0.007*			
Intralobular Inflammation	0.058	0.021-0.094	0.002*			
Ballooning Degeneration	0.090	0.039-0.140	0.001*			
Grade of fibrosis	0.080	0.044-0.116	<0.001*	0.054	0.017-0.090	**0.005***
Gender (male)	0.013	-0.043-0.070	0.638			
age	0.003	0.000-0.005	0.021*			
WC	0.002	-0.001--0.006	0.158			
BMI	0.031	-0.005-0.011	0.423			
DM	-0.017	-0.097-0.063	0.670			
Hypertension	0.047	-0.009-0.103	0.097			
AST	0.003	0.002-0.005	<0.001*	0.003	0.001-0.004	**0.001***
ALT	0.001	0.000-0.002	0.003*			

WC, waist circumference; BMI, body mass index; DM, Diabetes mellitus; AST, aspartate aminotransferase; ALT, alanine aminotransferase; *p<0.05

Sup Table S4 Factors associated with AC value

Characteristic	Univariate Analysis			Multivariate Analysis		
	Coefficient	95% CI	p value	Coefficient	95% CI	p value
Degree of steatosis	0.076	0.045-0.108	< 0.001*	0.077	0.038-0.116	**< 0.001***
Intralobular Inflammation	0.050	0.010-0.090	0.015*	0.008	-0.037-0.053	0.730
Ballooning Degeneration	0.068	0.012-0.124	0.019*	0.020	-0.046-0.087	0.546
Grade of fibrosis	0.013	0.005-0.021	0.001*	-0.032	-0.080-0.016	0.186
Gender (male)	-0.036	-0.097-0.025	0.245			
age	0.002	0.000-0.005	0.077			
WC	0.002	-0.001-0.006	0.160			
BMI	0.003	-0.005-0.011	0.492			
DM	0.090	0.008-0.172	0.031*	0.080	0.007-0.154	**0.032***
Hypertension	0.037	-0.024-0.097	0.233			
AST	-0.00001468	-0.002-0.002	0.986			
ALT	0.000007976	-0.001-0.001	0.982			

WC, waist circumference; BMI, body mass index; DM, Diabetes mellitus; AST, aspartate aminotransferase; ALT, alanine aminotransferase; *p<0.05

Sup Table S5 Factors associated with NLV value

Characteristic	Univariate Analysis			Multivariate Analysis		
	Coefficient	95% CI	p value	Coefficient	95% CI	p value
Degree of steatosis	-0.085	-0.128--0.042	< 0.001*			
Intralobular Inflammation	-0.073	-0.122--0.024	0.004*	-0.026	-0.051--0.001	**0.041***
Ballooning Degeneration	-0.063	-0.135--0.008	0.080			
Grade of fibrosis	-0.050	-0.103-0.003	0.064			
Gender (male)	-0.027	-0.140-0.050	0.485			
age	-0.004	-0.007--0.002	0.003*			
WC	-0.003	-0.005--0.001	0.007*	-0.002	-0.004--0.001	**0.012***
BMI	-0.011	-0.021--0.001	0.030*			
DM	-0.076	-0.181-0.029	0.151			
Hypertension	-0.104	-0.177--0.031	0.006*			
AST	-0.104	-0.003-0.001	0.323			
ALT	-0.001	-0.001-0.000	0.236			

WC, waist circumference; BMI, body mass index; DM, Diabetes mellitus; AST, aspartate aminotransferase; ALT, alanine aminotransferase; *p<0.05

Sup Table S6 Factors associated with NLV-SD value

Characteristic	Univariate Analysis			Multivariate Analysis		
	Coefficient	95% CI	p value	Coefficient	95% CI	p value
Degree of steatosis	-0.166	-0.257--0.075	0.001*	-0.060	-0.114--0.007	**0.026***
Intralobular Inflammation	-0.150	-0.253--0.047	0.005*			
Ballooning Degeneration	-0.147	-0.295--0.001	0.052			
Grade of fibrosis	-0.121	-0.231--0.011	0.031*			
Gender (male)	-0.041	-0.202-0.121	0.618			
age	-0.009	-0.015--0.003	0.006*			
WC	-0.007	-0.001--0.002	0.005*	-0.006	-0.011--0.002	**0.006***
BMI	-0.023	-0.044--0.002	0.032*			
DM	-0.164	-0.383-0.056	0.142			
Hypertension	-0.238	-0.389--0.087	0.002*			
AST	-0.002	-0.007-0.002	0.262			
ALT	-0.001	-0.003-0.001	0.247			

WC, waist circumference; BMI, body mass index; DM, Diabetes mellitus; AST, aspartate aminotransferase; ALT, alanine aminotransferase; *p<0.05

Sup Table S7 Factors associated with Ratio value

**Characteristic**	**Univariate Analysis**		
	Coefficient	95% CI	p value
Degree of steatosis	0.311	0.003-0.619	**0.048***
Intralobular Inflammation	0.213	-0.129-0.556	0.218
Ballooning Degeneration	0.448	-0.025-0.920	0.063
Grade of fibrosis	0.194	-0.164-0.553	0.283
Gender (male)	0.145	-0.368-0.657	0.575
age	0.011	-0.009-0.031	0.285
WC	-0.004	-0.035-0.026	0.775
BMI	-0.007	-0.075-0.062	0.849
DM	0.238	-0.469-0.944	0.505
Hypertension	0.244	-0.266-0.754	0.343
AST	0.011	-0.003-0.025	0.119
ALT	0.002	-0.003-0.008	0.408

WC, waist circumference; BMI, body mass index; DM, Diabetes mellitus; AST, aspartate aminotransferase; ALT, alanine aminotransferase; *$p<0.05$

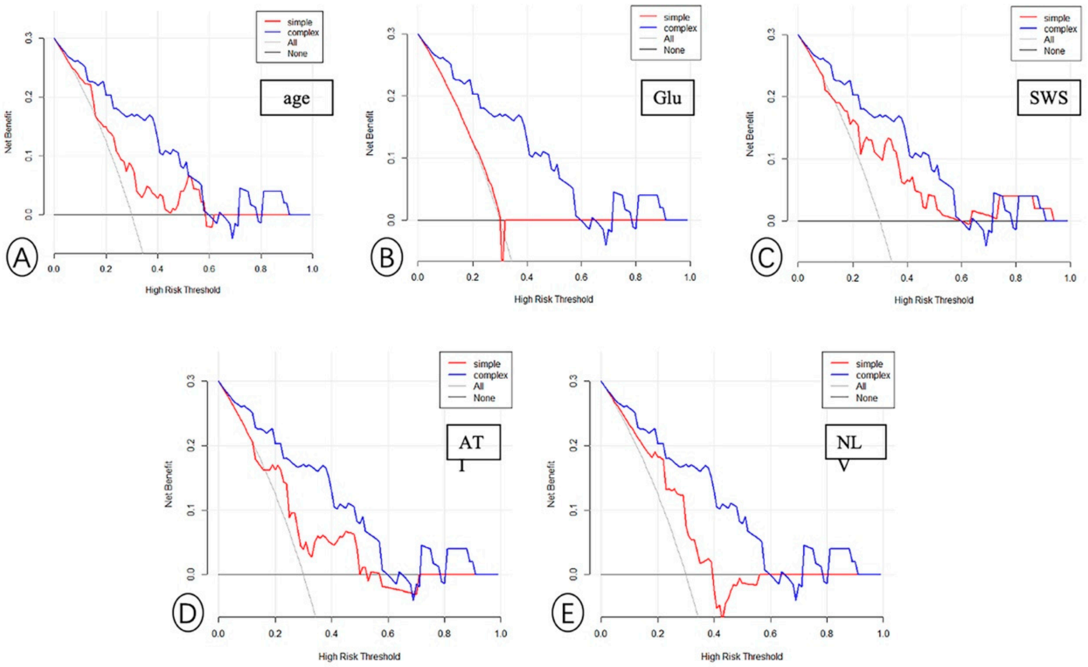

Sup fig S1. Decision curve analysis for the combined nomogram model. The y-axis indicates the net benefit; the x-axis indicates threshold probability. The red lines represent the net benefit of single parameters age (A), Glu (B), SWS (C), ATI (D), NLV (E), respectively. The blue lines represent net benefit of the combined nomogram model. The combined nomogram model has a higher overall net benefit than the single factor model within most reasonable threshold probabilities for the identification of NASH.
